# Cranial Cruciate Ligament Rupture in Dogs: Review on Biomechanics, Etiopathogenetic Factors and Rehabilitation

**DOI:** 10.3390/vetsci8090186

**Published:** 2021-09-06

**Authors:** Giuseppe Spinella, Giulia Arcamone, Simona Valentini

**Affiliations:** Department of Veterinary Medical Sciences, University of Bologna, Ozzano dell’Emilia, 40064 Bologna, Italy; giulia.arcamone@studio.unibo.it (G.A.); simona.valentini@unibo.it (S.V.)

**Keywords:** cranial cruciate ligament, rupture, biomechanics, etiopathogenesis, rehabilitation

## Abstract

Cranial cruciate ligament (CrCL) rupture is one of the most common orthopaedic conditions in veterinary medicine. CrCL plays a fundamental role in the stability and biomechanics of the femoral-tibio-patellar joint, and its incorrect functionality severely impacts on the quality of life of patients. In dogs, the structural weakening of this joint due to the progressive degeneration of the ligament is the most accredited etiopathogenetic hypothesis in relation to the dog signalment (breed, sex and age) and the stifle joint conformation. In humans, this injury is often traumatic and generally occurs during sporting activities. CrCL rupture can be managed conservatively or surgically, and decisions regarding treatment are due to numerous factors: the patient’s age and health, the degree of stifle instability, and cost. Physiotherapy protocols play an important role in rehabilitation, with similar goals in humans and dogs: pain management, physiological articular range of motion recovery, periarticular and core muscle strengthening, and proprioceptive deficit correction. Physiotherapy, even if often neglected in veterinary medicine, is mandatory for the recovery of the correct functionality of the injured limb and for the return to normal daily and sporting activities.

## 1. Introduction

Cranial or anterior cruciate ligament rupture is one of the most common orthopaedic diseases in veterinary and human medicine [1,2,3,4,5,6,7,8]. Witsberger et al. reported a prevalence of about 11% in the canine population referred to twenty-seven North American hospitals in the decade of 1994–2003 [9]. In humans, the incidence increased from about 90,000 cases in 1994 to about 130,000 in 2006 [10]; however, other authors place this number at closer to 200,000 cases [11,12,13]. Cruciate ligament inefficiency has important negative consequences on the quality of life of patients because it causes stifle instability, leading to the onset and progression of severe degenerative changes that can limit normal daily activities. In athletes, this injury may represent a concrete obstacle for the progression of their career: it has been shown that only 60% of dogs participating in agility competitions are able to return to compete after a Tibial Plateau Leveling Osteotomy (TPLO) surgery [14]; in human medicine, even if 80% of patients are able to return to sports, only about 60% of these patients achieve their pre-injury level of performance [15]. The present paper is a review of the scientific literature on the main etiopathogenetic hypotheses, the predisposing factors to cranial cruciate ligament (CrCL) rupture, and the principles of physiotherapy rehabilitation, underlining the main differences and similarities between the canine and the human species. The literature search was performed using specialized books and following online scientific databases: PubMed and Scopus. The studies were identified using different combinations of the following terms as keywords: cranial cruciate ligament, anterior cruciate ligament, injury, rupture, biomechanics, etiopathogenesis, predisposing factors, rehabilitation, dog, canine, and human.

## 2. Biomechanics

In both dogs and humans, the femoral-tibio-patellar joint, due to its strategic position between the hip and hock/ankle, plays a key role in the pelvic limb. In the standing position, thanks to the menisci interposed between the femoral and tibial condyles, the stifle joint absorbs and supports the bodyweight, while during the movement, it allows the transmission of the propulsive thrust towards the coxo-femoral joint and the shortening of the functional length of the pelvic limb [16,17,18,19]. Strong knowledge of the biomechanical proprieties of the stifle joint is essential for explaining its static and dynamic role as well as the etiopathogenetic hypotheses and therapeutic options in case of CrCL injury.

The stifle joint is marked out by six free degrees of movement around three planes: sagittal (flexion/extension and cranio-caudal translation), transverse (extra/intra-tibial rotation, and mid-lateral translation) and frontal (adduction/abduction and ventro-dorsal translation) [20]. Within this context, in both dogs and humans, the cruciate ligament is responsible for these peculiar activities: to limit stifle hyperextension and tibial intra-rotation on its axis and to hinder the caudo-cranial translation of the tibia in relation to the femur [21]. According to the traditional (passive or static) biomechanics model of the femoral-tibio-patellar joint, the intra-articular and peri-articular structures are liable for the stability of the knee, and the only movement allowed is flexion–extension on the sagittal plane [22,23]. In the active model, joint stability is the result of the balance between the action of the intra-articular and periarticular structures and the forces acting on the knee: the ground reaction force and the forces deriving from muscle contraction. During weight loading, these forces, resulting from the caudo-distal inclination of the articular surface (tibial plateau) [24,25], generate a cranially oriented shear force called cranial tibial thrust [26]. The cranial tibial thrust is gradually greater with the increase of the tibial plateau inclination, and it is passively counteracted by the CrCL [22,23,24]. In 2002 Tepic formulated a new biomechanics model according to which the direction and entity of the cranial tibial thrust and consequently the greater or lesser stress on the cruciate ligament change according to the angle between the patellar ligament and the tibial plateau. Cranial tibial thrust is neutralized when this angle is equal to 90° [27,28,29,30,31,32,33,34]. The origin of this model comes from human medicine. A previous study described the forces acting on the knee joint during weight loading and defined the resulting force as being approximately parallel to the patellar ligament, recording a cranial tibial thrust that is directed cranially or caudally depending on whether the knee is in the extended or flexed position [35]. The evolution of the biomechanical model of the femoral-tibio-patellar joint allowed the better understanding of the possible causes of the breaking point of the cranial cruciate ligament. The limits of the traditional model originate from the inability to justify the etiopathogenesis of specific CrCL injuries, such as rupture in the absence of hyperextension and in non-traumatic conditions. The explanation lies in the assumption that the forces acting at the level of the joint are in such a balance that they cannot damage the structures [23]. The most recent biomechanical models, on the other hand, show the existence of forces responsible for constant stress on the cruciate ligament, which can result in further weakening and rupture (partial or total). Tepic’s theory justifies that breeds with a greater predisposition to this injury are breeds with hyperextended pelvic limbs, such as the Boxer and Chow Chow, which results in greater stress on the ligament, which is also determined by the increased functional inclination of the tibial plateau [36,37]. Recent studies on gait analysis (kinetic and kinematics) have also allowed the development of current osteotomy techniques, which have the specific aim to neutralize the cranial tibial thrust and to restore the balance of forces that act on the stifle joint [22,23,25,33]. Kinetic and kinematic analysis allow the investigation of the role of the anatomical structures that compose the knee joint and its biomechanical properties [20,38,39]. Through specific force platforms, kinetic analysis allows the estimation of the forces acting at the limb level and, consequently, at the joint level. The forces acting at the joint level vary during the different phases of gait: they are at maximum effect during the stance phase, while they are at zero in the swing phase. The force platform allows the detection of even minimal variations of Ground Reaction Force (GRF), highlighting even the slightest lameness, which is difficult to identify during clinical examination [39,40]. Kinematic analysis allows the evaluation of how the position, speed, and angular excursion of the knee joint change during motion [38,41,42,43,44,45,46]. The kinetic and kinematic studies allow the variations that occur in case of CrCL rupture compared to a normal gait to be highlighted. Throughout the gait cycle in a dog, in the case of CrCL rupture, the stifle generally appears more flexed, while the hip and tarsal joints appear more extended during the stance phase as a compensatory effect [25,47,48,49,50]. The decrease in the stifle extension is assumed to represent an adaptive expedient used by the dog to reduce limb load, pain, and instability [47,49]. The flexion of the limb, on the other hand, mobilizes the hamstrings, resulting in a condition that would tend to stabilize the tibia and prevent cranial translation. In most dogs with CrCL rupture, there is also a greater cranial subluxation of the tibia to the femur during the limb stance phase, while its position remains almost unchanged in the swing phase [25,47,49,50]. Korvick has shown that the displacement of the tibia occurs during the activation of the quadriceps muscle which generates, together with body weight and inclination of the tibial plateau, the shear force that pushes the tibia in the caudo-cranial direction. Since both the activity of the quadriceps and the gravitational forces are minimal during the swing phase, the cranial thrust of the tibia is also lost [49]. Studies by Tashman and collaborators in 2004 also demonstrated that CrCL rupture does not cause substantial changes in external knee rotation in dogs but rather reduced internal rotation [25,49]. Considering that the main role of the cranial cruciate ligament is to prevent excessive tibial internal rotation, we would have expected an increase in the internal rotation following its rupture. The absence of truly significant changes in internal and external rotation suggests that these rotational movements are also affected by factors such as bone geometry, muscle forces, and soft tissue constraints other than the cranial cruciate ligament [49]. Differently, in a study by Tinga and co-authors, a greater internal rotation of the tibia was observed in dogs with CrCL rupture, especially in the stance phase [50]. When the action of the cruciate ligament disappears, the collateral ligaments become the alternative containment system of the cranial subluxation of the tibia as well as represent the main containment of the rotational movements on the transverse plane. Consequently, dogs with CrCL rupture tend to keep the knee slightly flexed, and the lateral collateral ligament is not sufficiently stretched to oppose the sliding of the lateral condyle of the tibia in the cranio-medial direction, leading to the internal rotation of the tibia [50]. In addition to the kinematic alterations previously described, kinetic analysis allows the detection of an evident decrease in the vertical peak forces and impulses during the support and propulsion phase, testifying to the patient’s reluctance to load weight on the injured limb, with a frequent mild lameness during clinical examination [25,47,51,52]. As demonstrated in several studies, three-dimensional kinematics of knees with anterior cruciate ligament (ACL) deficiency has led to modifications in human medicine. Similarly to dogs, in injured patients, an increase in tibial intra-rotation [53,54], an increase in knee flexion [54,55], and an increase in the anterior translation of the tibia [56] were recorded. Regarding the last point, in other studies, no significant differences were found compared to healthy patients and, according to the authors, this was justified by the ability to compensate for the inefficiency of the ligament through the lower activation of the quadriceps muscle with greater activity of the posterior limb muscles, as confirmed by electromyographic studies [54,55,57].

## 3. Etiopathogenetic Hypotheses

The exact etiopathogenesis of cruciate ligament rupture in dogs and humans has not been fully clarified. In the past, CrCL rupture was believed to result almost exclusively from a traumatic event, but over the years, it has been shown that this disease arises also spontaneously during physiological load due to degenerative changes in the stifle joint [2,3,5,58,59,60]. However, accidental traumatic injury represents a possible cause of acute rupture, which can occur in dogs of any breed and any age [60]. In human literature, the non-traumatic origin of ACL rupture is not well described. In most cases, men show this disease during sudden changes of direction and/or landings or due to impact with opponents [5,8,61]. The degenerative changes occurring in the cranial cruciate ligament are responsible for a progressive reduction of its elasticity and mechanical resistance, which make it more susceptible to even minimal trauma or, ultimately, to the stresses arising from the physiological daily load on the stifle. Degeneration, chondroid metaplasia, and the decrease of fibroblasts as well as the loss of the normal organization and orientation of collagen fibers have been observed. Ichinohe and co-authors analyzed the injured and intact cranial cruciate ligaments of two groups of dogs from a histological and immunohistochemical point of view, evaluating the different expression of type I collagen (COL-I), type II collagen (COL-II), collagen type III (COL-III), and Serotype HMG box 9 (SOX-9), a chondrocyte-specific transcription factor that regulates the chondrogenic differentiation of mesenchymal cells and the transcription of chondrocyte-specific genes. In patients with CrCL, a low expression of COL-I and a greater expression of COL-II and COL-III were observed; in addition, there was a high number of fibroblasts with perinuclear halos with characteristic SOX-9 positivity. The reduction of type I collagen justifies the lower tensile strength of degenerated ligaments, while the presence of type II collagen and SOX-9 is indicative of chondroid metaplasia fibroblasts [62]. The human cruciate ligament undergoes cellular and vascular composition changes that alter biological characteristics, healing capacity, and biomechanical function [63]. However, a histological study on cadavers by Hasegawa and collaborators showed that the main change that occurs in the human ligament was the loss of the typical collagen fiber organization [64]. Studies on degenerative changes in the cranial/anterior cruciate ligament of dogs and men suggest structural changes resulting from ligament remodeling and physiological adaptation to the stress deriving from mechanical load, micro-injuries related to conformational anomalies, and hypoxic conditions due to poor blood flow, which are more evident in the central part of the ligament [3,24]. Many of these changes are physiological and appear to be associated with aging [3,65,66]. However, even if age is one of the predisposing factors for spontaneous CrCL rupture, other elements are needed to justify the onset of this injury because its incidence would be higher than hypothesized [3]. Some authors believe that inflammation could arise in the knee joint before CrCL rupture and that these inflammation processes are capable of altering the composition of the ligament and reducing its mechanical resistance [67,68]. Many studies support the hypothesis that these inflammatory phenomena are immune-mediated: thus, in the synovial fluid of injured patients, a high titer of auto-antibodies against type I collagen has been observed [2,69,70,71,72,73]. Cruciate ligaments are covered by a fold of the synovial membrane, but they are extra-synovial, isolated from the surveillance of the immune system. In case of injury, the exposure of their collagen can act as a self-antigen, triggering the inflammatory phenomenon and the release of antibodies and macrophages [2,58,74]. de Rooster and collaborators hypothesized that antibodies against COL-I do not play an active function in the onset of ligament damage, but they are responsible for its perpetuation [73]. However, de Bruin and collaborators demonstrated that not all dogs with high titers of auto-antibodies in the synovial fluid undergo CrCL rupture, suggesting a non-specific role in joint disorders [75]. The role of auto-antibodies must therefore be further investigated and considered together with other etiopathogenetic factors. The chemical mediators of inflammation can also have an indirect role in progressive ligament degradation. As is known, the structural properties of the cruciate ligament depend on the composition and nature of the extracellular matrix and depend on the collagen whose turnover derives from a careful balance between its synthesis and degradation in particular. In this mechanism, proteases such as cathepsins and matrix metalloproteinases (MMPs) including collagenases (MMP-1, MMP-8, and MMP-13), stromelysins (MMP-3 and MMP-10), and gelatinases (MMP-2 and MMP-9) play a very important role [76,77,78,79,80]. In two distinct populations of Labrador Retrievers, it has been shown that the concentration of MMP-2 is significantly higher in dogs with a broken cruciate ligament than in subjects with an intact ligament [81]. A further study demonstrated the existence of a precursor of MMP-2 (pro-MMP-2) that is higher in the cruciate ligaments of high-risk breeds (Labrador Retrievers) than in low-risk breeds (Greyhounds) [82]. The results of all of these studies suggest that in the damaged ligament, there is a greater turnover of the extracellular matrix, which can be triggered by inflammatory phenomena and could lead to a reduction in strength and cause a consequent rupture [80]. Ligament remodeling is also influenced by two other factors: tartrate-resistant acid phosphatase (TRAP) and cathepsin K. Tartrate-resistant acid phosphatase (TRAP) belongs to a group of iron-binding proteins and is able to mediate the process of collagenolysis. Cathepsin K, on the other hand, is a proteinase capable of determining the lysis of type I collagen. Their overexpression in subjects with CrCL rupture suggests how this may contribute to the progressive structural failure of the ligament [77,78]. It is important to underline that significantly greater amounts of TRAP and cathepsin K were found in the canine injured ligament than in those in human ones, suggesting a different contribution of proteolytic phenomena to the etiopathogenesis of cruciate ligament rupture in these two species [78].

## 4. Predisposing Factors

The main predisposing factors for cruciate ligament rupture are divided into biological and biomechanical factors. Biological factors include breed, sex, age, and genetics while biomechanical factors include stifle joint conformation, the alignment of bone segments, and muscle strength [59,60] (Table 1). All of these elements contribute to the acceleration of the degenerative changes that occur within the joint and the cruciate ligament, justifying the considerable differences in injury incidence in different breeds although the disease can actually occur in patients of any breed, sex and age.

### 4.1. Breed and Body-Weight

Cranial cruciate ligament rupture is a very common condition in large and/or overweight dogs. In these patients, the cranial cruciate ligament undergoes degeneration more easily, as the joints are subjected to greater stresses that can clearly accelerate and favor the degradation processes [83]. The main dog breeds affected by this injury are Labrador, Newfoundland, Rottweiler, Neapolitan Mastiff, Saint Bernard, Chesapeake Bay Retriever, American Staffordshire Terrier, Akita, Boxer, and Bulldog [2,3,9,24,25,59,84,85]. In these breeds, it is also possible to observe anomalies that can contribute to cruciate ligament weakening and rupture, such as, for example, the hyperextended position of the pelvic limbs with highly open joint angles [37,86], which occurs frequently in breeds such as the Chow Chow, Rottweiler, American Staffordshire Terrier, Boxer, and Saint Bernard. Hyperextended pelvic limbs are often associated with hip dysplasia, which is recorded in a high percentage of dogs with CrCL rupture [2,9]. Although cranial cruciate ligament rupture is more likely to occur in large dogs, this does not exclude that such injury may also affect small breeds. In most cases, in small dogs, ligament injury occurs following excessive stress caused by tibial instability associated with patellar luxation (especially grade IV) [5,87,88]. In these breeds, the ligament is also stressed by genu varum, an altered conformation of the pelvic limbs [86,89]. Another predisposing factor is obesity, in which the concentrations of circulating inflammatory mediators increase, such as the concentrations of pro-inflammatory adipokines released from adipose tissue, which may contribute to degenerative phenomena and ligament weakening [90,91,92,93]. Hypotheses on the role of adipose tissue in the etiopathogenesis of ACL rupture have also been advanced in humans. Adiponectin is an adipokine encoded by the “ADIPOQ” gene and is secreted into the circulation by the adipose tissue: it has many biological functions, including the regulation and the production of matrix metalloproteinases (MMPs), which are mainly responsible for the remodeling of the extracellular matrix of ligament tissues. For this reason, adiponectin has been proposed as a possible mediator of cruciate ligament disease [94]. In a recent study, Udomsinprasert and collaborators demonstrated that the genetic polymorphism linked to the production of adiponectin represents a risk factor in the onset of this lesion, focusing their attention on the single nucleotide polymorphism (SNP) localized in 276 pairs of bases downstream of the gene encoding the hormone (276 G/T). According to the results of this study, the risk increases in homozygous patients with the G allele (genotype GG) and occurs more generally in subjects expressing the G allele, unlike subjects homozygous for the T allele (genotype TT). In effect, the GG genotype suppresses the transcription and expression of adiponectin, causing an imbalance in the synthesis and degradation of the extracellular matrix of the ACL and therefore its weakening [94].

Another important consideration is related to a sedentary lifestyle in obese subjects, both in the canine and human species. Inactivity contributes to the weakening of the ligament and the periarticular soft tissues (especially muscles and tendons), which protect and stabilize the joint in the static phase and during a wide range of dynamic movements. Inadequate muscle development and aberrant neuromuscular control are thought to adversely affect knee stability, contributing to increased stress on its ligament components and progressive cruciate ligament degeneration [95]. In humans, the role of correct neuromuscular activation in the prevention of stifle soft tissue injuries has been studied extensively. The recruitment of an insufficient number of muscle fibers increases the possibility of ACL injury, especially in the case of sudden changes of direction. The sources of proprioceptive information reside primarily in the mechanoreceptors located in the periarticular soft tissues and in the cruciate ligament itself [96]. Therefore, there are specific preparation programs and proprioceptive training aimed at implementing these mechanisms and conditioning the muscle for correct activation in the prevention of injuries and post-operative rehabilitation. Caraffa and collaborators have shown how a targeted training program, which was tested on professional footballers, is able to significantly reduce the incidence of injuries to the ACL [97].

### 4.2. Age

Cranial cruciate ligament injury is common in the age range of 2–10 years of age [9,84,85,98,99]: in particular, dogs of less than 4 years of age generally undergo traumatic rupture, while in dogs between 5 and 7 years of age, spontaneous rupture following degeneration has been more frequently observed [3]. Age has to be also correlated with weight: in small-sized dogs, the lesion occurs in the oldest ones as degenerative phenomena affecting the ligament occur later since the lower weight limits the alteration of the elastic and mechanical strength of the ligament [3]. Acute traumatic rupture can occur in subjects of any age; nevertheless, it is assumed that this injury mainly affects young subjects, as they are more active and more likely to be employed in recreational and sporting activities, with the possibility of making abnormal movements and with greater stress on the knee joint. In young dogs with an immature skeleton, it is possible to find the avulsion of the ligament from its femoral more frequently or, more commonly, from tibial insertion site since the terminal fibers inserted in the bone are stronger than the bone itself [25]. In humans, injury is mainly concentrated in people who are between 15 and 45 years of age, as they are more likely to be physically active [6,96]: the wide age range is justified by the traumatic etiology of this injury, which is generally secondary to a sporting activity [96]. According to a study by Butler conducted in 1989 at the University of Cincinnati in Ohio, people in this age group were more likely to be referred for knee joint injuries, and in 70% of these cases, the ACL was completely or partially disrupted [100].

### 4.3. Sex

Whitehair and collaborators pointed out a higher incidence of cranial cruciate ligament rupture in female dogs rather than in males [84] This observation has been partially confirmed by other studies, which showed a greater predisposition to CrCL rupture in neutered males and spayed females [2,4,9,24,59,98,101,102,103]. The origin of gender difference is multifactorial, and it has also been associated with the stages of the reproductive cycle. In human medicine, some authors agree in defining the ovulatory phase where patients are the most at risk, highlighting how high levels of estrogen reduce the production of collagen and therefore influence the size and resistance of the ligament [104,105]; other authors recognize a higher incidence during the initial follicular phase [106]. The discrepancy between the results of these studies and the considerable variability of total cycle length and different phases as well as sex hormone levels suggest that further investigation is needed to determine which phase of the cycle places patients the most at risk for onset of cruciate ligament injury [107]. Connective tissue remodeling and metabolism are the result of the continuous synthesis and degradation of proteins: this process is also regulated by steroid hormones. Therefore, the alteration of the hormonal structure resulting from ovariectomy and orchiectomy can determine a greater predisposition to ligament rupture [102,108,109]. Receptors for the luteinizing hormone (LH) have been found inside the ligament: in spayed and neutered animals, an increase in the activation of these receptors results in increased CrCL laxity, making it more susceptible to the onset of lesions. Furthermore, prepuberal spaying and neutering delay tibial growth plate closure, resulting in an increase of the tibial plateau caudo-distal inclination with an increase in cranial tibial thrust, a risk factor for CrCL rupture [108,110]. Sterilization and castration also have an indirect role in the incidence of the injury, as they can be related to an increase in body weight and obesity. However, it should be emphasized that the reduction in hormone levels also induces important behavioral changes, making animals less aggressive and limiting the risk of injury [24,103]. In human medicine, several studies have shown that women show a 4 to 6 times greater predisposition to this type of injury than the male population [6,111,112]. In addition to hormones, it is important to indicate that this gender difference is due to many other factors that can be classified into anatomical, biomechanical, genetic, and social [61,113].

### 4.4. Genetics

Some authors have hypothesized the existence of a genetic component in the risk of CrCL rupture [5,59,60,114,115,116,117,118,119]. In 2006 study on the Newfoundland breed, Wilke and collaborators demonstrated that a single recessive gene with incomplete penetrance, for which a correlation with the structural properties and mechanical strength of the ligament is hypothesized, is responsible for this injury [114]. Three years later, the same authors, analyzing the genome of dogs of the same breed, found the presence of markers called microsatellite markers (MSATs) on chromosomes 3, 5, and 13, which they determined to be associated with cranial cruciate ligament rupture [115]. The results of this study were only partially confirmed by Baird and collaborators, who identified markers associated with CrCL rupture on chromosomes 1, 3, and 33 and in particular at the level of the genes involved in the transmission and modulation of the nerve impulse towards the articular and periarticular structures. The alteration of these genes may be responsible for a predisposition to this injury, as an abnormal nervous transmission can determine the reduction of proprioceptive abilities which, in both dogs and humans, can expose the cruciate ligament to a greater risk of injuries [117]. In the same year, the authors identified single nucleotide polymorphism (SNP) affecting some of the genes responsible for the strength and structure of the cruciate ligament (COL-1A1 and COL-5A1 collagen genes), formation and organization of fibrils and ligament elasticity (LTBP2 gene or Latent Transforming Growth Factor Beta Binding Protein 2), formation of the extracellular matrix (fibrillin FBN1 gene), and collagen cross-linking (LOX gene of lysis oxidase) suggesting an important role in the greater or lesser susceptibility of different breeds to cranial cruciate ligament rupture [116]. More recent studies have identified in some breeds, such as Newfoundland and Labrador, single nucleotide polymorphism (SNP) affecting the genomic loci responsible for the angulation of the tibial plateau and the width of the tibial tuberosity, both of which are risk factors for CrCL rupture [120]. Similar to what has been described in dogs, in humans, several studies have suggested that there may be a genetic basis at the onset of the disease. A familiar predisposition with mutations in collagen genes, particularly COL-1A1 [121] and, especially in women, COL-5A1 [122,123] and COL12A1, were observed [116,118,124,125]. Furthermore, in all likelihood, alterations in the LOX gene can also contribute to the onset of lesions in the ACL, as they are found in subjects suffering from Ehlers–Danlos syndrome, which is characterized by the alteration of collagen and ligament laxity [116].

### 4.5. Stifle Deformation

In both species, it is possible to identify some particular knee conformations that can increase the risk of injury to the cruciate ligament; in human medicine, knee conformation also contributes to justifying the great difference in predisposition between the two genders, as they are more likely to be found in female subjects. The most studied issues are the size and shape of the intercondylar notch and the inclination of the tibial plateau. Comerford and collaborators, in a comparative study between Labradors and Greyhounds, showed that the size of the intercondylar notch is significantly smaller in the first breed and involves a particular predisposition to CrCL rupture [82]. The most accredited hypothesis is that the narrowing of the intercondylar notch causes an increase in the contact surface between the ligament and the notch itself. Repeated stresses of the ligament cause an increased collagen metabolism and turnover: in subjects with a narrow intercondylar notch, greater expression and activity of the collagenolytic metalloproteinases, in particular MMP-2, which is responsible for progressive degeneration and consequent CrCL rupture, has been observed [82]. The narrowing of the intercondylar notch may be due to a primary congenital anomaly or be secondary to degenerative changes that occur within the joint. An increase in load can alter joint mechanics, contributing to intercondylar notch remodeling, and narrowing peculiarity was frequently observed in Labradors, a breed that is particularly predisposed to obesity [126]. In human medicine, many authors have highlighted how, in both sexes, the reduced development of the intercondylar fossa determines an increase in the probability of ACL rupture; however, they also agree that these anomalies are more frequent in women than in men [61,127,128,129,130]. Many authors have associated the predisposition to this injury with an increased tibial plateau inclination, which results in greater cranial tibial thrust [58,131]. The results of some studies suggest that the increase in the tibial plateau inclination may be the result of early sterilization [108,110]. However, opinions regarding the contribution to ligamentous degeneration of this particular shape of the tibia are currently much debated. Although it is possible to find a different tibial plateau inclination in breeds with different predispositions to CrCL rupture [132], some authors have shown that there are no significant differences in subjects with and without CrCL rupture [133]. A more important role has been attributed to the shape of the tibial tuberosity and to the angle between the tibio-patellar ligament and the tibial plateau [134]. Additionally, in human medicine, especially in women, the inclination of the tibial plateau represents a risk factor for this injury [61,135,136,137]: a very important role is also played by the abnormal valgus knee conformation that alters joint biomechanics and forces distribution at this level, justifying the greater predisposition to CrCL rupture seen in women [8,19] (Table 2).

### 4.6. Traumatic Injury

Traumatic rupture of the cruciate ligament is very common in sportive dogs and men. It usually occurs following the hyperextension of the knee [5,36,138], excessive intra/extra tibial rotation [5,138], or excessive cranial tibial thrust [5,138], which can occur when performing sudden changes of direction or when landing from high altitudes. In human medicine, these movements, together with traumatic impact with opponents, are recognized by numerous authors as the main factors that are responsible for a lesion of the ACL [6,139,140]. Cruciate ligament rupture occurs when a change of direction is performed incorrectly. The factors for incorrect execution and increased knee valgus stress are hip adduction and internal rotation, tibial external rotation, and external rotation and eversion of the ankle [6,141]. These conditions cause stress on the ACL and may be more force than it can withstand, often resulting in a traumatic rupture. Ford and collaborators demonstrated that, when performing changes of direction, the degree of knee abduction in women was significantly greater than it was in the male control group, further explaining the greater predisposition to this injury in females, which is also justified by a different strategy during the landing phase from a jump [142,143]. In this case, the main risk factors for ligament injury are an extended and internally rotated hip [144], reduced knee flexion and abduction, and internal rotation of the tibia [6]. In most cases women land in a more upright position and with a greater knee extension and abduction, which causes a greater posterior–anterior translation of the tibia. In contrast, men land with a semi-flexed knee, and this position is more suitable for shock absorption and energy transfer throughout the pelvic limb [113,142,145]. In veterinary medicine, changes of direction and landing from high altitudes represent a risk, especially for dogs participating in agility and disc dog competitions [146,147,148,149]. Even dogs not engaged in specific sports can exhibit a traumatic CrCL rupture during a jump or run. This occurs to a greater extent in dogs who are inactive during the week and very active during the weekend: this condition is known as “weekend warrior syndrome” [138]. The limited physical condition and poor muscle tone typical of these dogs can be decisive in the onset of CrCL rupture. Muscle development is essential for knee protection: the degeneration and rupture of the ligament can arise in these dogs as a result of an imbalance between the flexor and extensor muscles of the thigh, similar to what is described in humans [84,85,150].

## 5. Rehabilitation

Nowadays, physical rehabilitation in veterinary medicine represents an integral and fundamental part of protocols for the management of common neurological and orthopedic diseases, including cruciate ligament rupture [151,152]. The therapeutic options in case of CrCL rupture can be divided into two categories: surgical and conservative. Surgical treatment represents the gold standard for injury correction, as it is more effective in correcting instability, restoring joint function, and delaying the onset of osteoarthritis [153]. In 2013, Wucherer and collaborators investigated the long-term outcomes of surgical and non-surgical treatments in two groups of overweight dogs with unilateral cranial cruciate ligament rupture. The assessment was made on the basis of a questionnaire provided to the owners to express their perception of the improvement in lameness; in addition, the degree of lameness was objectified through the use of force platforms. The results of this study confirmed that dogs undergoing surgery along with a specific diet to lose weight, the administration of nonsteroidal anti-inflammatory drugs (NSAIDs), and a specific rehabilitation protocol showed better overall results than patients treated conservatively [154]. Conservative management can be chosen by the owner for many reasons: individual preference, the activity level and age of the dog, concomitant serious diseases, and economic and financial reasons [155,156,157,158]. In a study conducted in 2005, Wilke and collaborators demonstrated that the economic impact of cranial cruciate ligament rupture is significantly higher in cases where surgery is performed due to many factors, including the skill level of the surgeon, the surgical technique, and the cost of the material [11]. In contrast, in human medicine, although the costs for the surgical resolution of the ligament rupture are high, some authors point out that the economic impact of this injury is greater in cases where conservative management is chosen due to the higher costs for the management of post-traumatic osteoarthritis and the indirect costs related to lost patient wages, inactivity, and disability [12]. The choice to treat a dog conservatively or surgically also depends on the weight of the patient, the stability of the joint, and the severity of the clinical signs [156,159]. Conservative management may be effective in small-sized individuals (<15 kg) and/or individuals with partial ligament rupture; however, there is a high probability that this will progress to a complete rupture over time [11,83,155,158]. In a 1985 study by Vasseur, 75% of 80 dogs treated with conservative management and weighing less than 15 kg were clinically normal over a three-year follow-up, compared to only 7% of patients weighing more than 15 kg [83]. In human medicine, conservative treatment is generally applied in elderly or more sedentary patients, considering that the knee retains a certain degree of instability and is predisposed to degenerative lesions [160]. In human medicine, patients who need to return to sports (such as football, basketball, and skiing) are always surgically treated [161].

Rehabilitation can play a pivotal role in restoring the functionality of the injured limb [162,163,164,165,166,167]. Regardless of the therapeutic option, the objectives of the rehabilitation protocols are pain management, recovery of normal joint kinematics, periarticular and core muscles strengthening, and correction of proprioceptive deficits [156,158,159,162,166,168,169,170,171,172,173]. When the surgical approach is chosen, the therapist has to know the technique that is to be used in order to plan the most suitable rehabilitation protocol for the different phases of tissue healing [173]. A fundamental aspect of the rehabilitation protocol is to limit prolonged disuse of the limb in order to avoid negative effects such as muscle atrophy, reduced joint mobility, cartilage atrophy, loss of strength of tendons and ligaments, and osteopenia [158,169,170]. Following extracapsular surgical stabilization, activity restriction is recommended for the first 8 weeks after intervention in order to not interrupt the process of periarticular fibrosis, which is essential for joint stability. However, numerous studies highlight the benefits of starting rehabilitation activities at an early stage [173]. In a 2002 study, Marsolais and collaborators compared the outcomes of different post-operative management in two groups of dogs undergoing extracapsular surgical stabilization: one group underwent a period of activity limitation after surgery only, and the other group was submitted to a specific post-operative rehabilitation protocol. After 6 months, the peak of vertical force (PVF) on the affected limb was recorded using force platforms in dogs from both groups. The PVF values were significantly higher in the dogs in rehabilitation [168]. If an intracapsular technique has been performed, the therapist has to consider the material used for the surgery. Between the 2nd and 20th weeks post-surgery, the graft is weak as it goes through revascularization and bio-integration processes. Therefore, even if rehabilitation is very similar to that for extracapsular techniques, it is imperative to proceed gradually [170,171]. Greater caution is also taken in the management of patients undergoing osteotomy because there are greater risks of post-operative complications. In addition, about 8–12 weeks are required for bone healing; however, most dogs are able to load weight on the affected limb fairly early [173]. Therefore, many authors agree that the tendency to prescribe absolute rest in the immediate post-operative period is currently overcome and replaced by targeted physiotherapy in order to guarantee excellent chances of the dog returning to its normal activities to protect its quality of life [174,175]. Pain management, in addition to specific pharmacological protocols mainly involving the use of NSAIDs, is based on cryotherapy, transcutaneous electrical stimulation (Transcutaneous Electrical Nerve Stimulation: TENS), and laser therapy [155,158]. Cryotherapy is not only used during the tissue damage phase, but also at the end of each rehabilitation session [176,177,178] and in the post-operative period [169,172]. In 2010, Rexing and collaborators demonstrated the efficacy of low temperatures in reducing pain and post-operative edema in subjects treated by means of extracapsular stabilization [179]. Other authors have proved that in the first phases following an osteotomy for leveling the tibial plateau, cryotherapy allows pain and swelling to be reduced and improves stifle mobility [180,181]. Transcutaneous electrical nerve stimulation (TENS) is also an effective method for pain management. With force platforms, Levine and collaborators recorded the ground reaction forces relating to the limb affected by the CrCL rupture before and after transcutaneous electrical stimulation, finding a significant improvement in the values in the second situation. They demonstrated how this treatment can reduce the pain secondary to osteoarthritis, a common consequence of ligament resection [178,182,183]. Another technique for pain control is laser therapy [178,184,185]. The role of laser therapy in pain modulation is justified by a direct effect on the peripheral nociceptors, which is secondary to the release of endorphins and enkephalins and to the blocking of pain signal transmission to the brain. Laser therapy also has an anti-inflammatory effect, as photons are able to determine the reduction in the concentration of prostaglandin E2 (PGE-2) and cyclooxygenase-2 (COX-2) [178,184,185]. Similar to TENS, for laser treatment, is shaving the hair in the area of application is recommended in order to increase the efficacy of this treatment [178]. Despite being one of the most used techniques in physiotherapy for post-operative pain management [186], some authors have raised some doubts the real effectiveness of laser treatment when used during a therapeutic approach for CCRL [187]. In 2018, a study was performed on dogs submitted to TPLO surgery, and two groups were created (only one underwent laser therapy). During the weeks following treatment, the owners completed a questionnaire about their impressions of their dogs’ pain and lameness improvements. The laser group apparently did not show any benefit in terms of pain reduction and inflammation reduction when compared to the control group [187]. Conversely, Rogatko and collaborators demonstrated the usefulness of laser therapy before osteotomy surgery because this technique provides protection against stress and tissue damage caused by surgery, reducing inflammation and increasing analgesia and vascularity. In the postoperative period, this resulted in faster functional recovery, as evidenced by significantly higher values of ground reaction forces than in the control group. However, no significant effects were found during radiographic examination and in the lameness degree at 8 weeks after surgery [188]. Another main goal of the physiotherapy rehabilitation program is the restoration of the normal ROM of the stifle joint. Therapeutic exercises for joint mobilization are highly recommended in order to counteract the negative effects of the disuse and immobilization of the limb. Passive exercises are conducted by the therapist on all of the joints of the injured pelvic limb (from hip to digit joints), while the active ones may be assisted (when the therapist supports the patient during the execution of the exercise) or not assisted (when the patient gains more autonomy) [185,189,190,191,192]. In addition, it is possible to perform some movements defined as accessories, which are fundamental for the restoration of normal joint function, such as distraction, compression, rotation, and sliding in the cranial and caudal direction of the tibia in relation to the femur [190,192]. Active exercises include some activities that are capable of soliciting angular excursion: mobility exercises in the pool, walking with obstacles, sitting to standing, and walking on a treadmill. In the first phase of rehabilitation, the assistance of the therapist is essential, especially in patients who are more reluctant to move, in order to encourage them to use the injured limb [156,189,193,194,195]. During all of these activities the therapist guides the patient and supports them to reduce the load on the joint. Depending on the size and weight of the animal, the therapist can lift it manually or with a harness (thoracic, abdominal, or pelvic). Alternatives to harnesses and manual support are simple elastic bands or towels placed under the dog’s abdomen [189,196]. Dogs who are capable of walking independently can also be introduced to more complex and intense active exercises without the assistance of the therapist. All of these activities are fundamental, as they correct atrophy and increase the tone of the muscles responsible for the stability of the stifle. As demonstrated in a study by Millis and collaborators, after 5 weeks from the cranial cruciate rupture, the circumference of the thigh decreases significantly if no targeted intervention is undergone [166,197]. When an approximately normal level of limb function is reached, it is advisable to increase the duration, intensity, frequency, and difficulty of the exercises so that patients reach adequate and optimal muscle tone to support the body and for the correct execution of the movement [156,185,189,195,198]. For example, during all of the above activities, whether performed in water or not, the therapist can fasten some weights to the dog’s body or use elastic bands in order to increase the resistance to be overcome for the correct execution of the movement [198]. The strengthening of the periarticular muscles, especially in dogs who are more reluctant to exercise, can also be obtained by neuromuscular electrical stimulation (NMES); this technique is simultaneously effective in reducing edema and pain and in promoting joint mobility [183]. In a 2008 study, Pellizzari and collaborators demonstrated that NMES can increase the thigh circumference in dogs suffering from atrophy following knee immobilization [199], confirming the previous results obtained by Johnson and collaborators in 1997 [164,200]. To provide greater stability to the hindlimbs during normal daily activities and to prevent improper movement of the stifle joint, rehabilitation programs also include exercises aimed at strengthening the core muscles. Some typical exercises are going from a sitting to standing position on fit-ball or trampoline, rolling, crunching, super-pointing, sitting in a hup and prayer position, planking on ball, and standing [194,198,201]. In addition to the proprioceptive inputs derived from the activities described, there are specific activities aimed at recovering the coordination skills of the injured limb, and these can be later applied when the dog is able to adequately load the weight on the injured limb and is able to walk normally [156,195]. Simpler exercises consist of placing the dog on inflatable or foam rollers or on a fit-ball and implementing extremely delicate bouncing movements to provide proprioceptive inputs and to stimulate muscle contraction of the injured limb [190,196,201]. Alternatively, external force can be applied to the dog while walking in order to shift its center of gravity outside of the support base and to force the dog to restore balance [196]. More complex exercises involve the use of balance boards (proprioceptive oscillating tablets), trampolines, inflatable platforms, pillows and mattresses made of foam rubber, or proprioceptive hemispheres [169,196]. By putting these devices in series, it is possible to create real paths to correct the proprioception deficit (proprioceptive pathways). In the daily management of the patient, braces and stifle pads are often used. The aim is to provide mechanical support to the dog in order to reduce knee instability by neutralizing the cranial tibial thrust present during CrCL rupture. These devices, as well as offering protection against impacts, slow down movement and allow a greater control of the pace thanks to muscle activation. In addition, they improve proprioception and reduce fatigue by taking some of the load off of the injured limb, as demonstrated by Carr and collaborators [157,202]. Some exercises for the rehabilitation of CrCL rupture can be completed in specific pools. Over the years, aquatic therapy has become a valuable component for veterinary physical rehabilitation to promote muscle strengthening, ROM improvement, and pain reduction [203]. Aquatic therapy on an underwater treadmill is important in the early stages of rehabilitation, where the patient is more reluctant to load weight on the affected limb. The advantage is that in immersion, a greater active articular excursion is detected, and muscle strengthening is less tiring. Furthermore, by regulating the water level, the joint kinematics can be influenced to require mor or less force in order to overcome the surface tension of the water [203,204,205,206]. Bertocci and collaborators have demonstrated the importance of accurately choosing the water level of underwater treadmills in the rehabilitation of dogs undergoing extracapsular stabilization. Kinematics of the pelvic limb joints and the duration of the stance phase change depending on whether the water level is below the hock or above the hock, knee, or hip. When the water level increases, the ROM in the sagittal plane of the three joints increases, and it is at its maximum when the limb is completely immersed. The results of this study suggest that joint function restoration is optimal when the water level is at or above the stifle [207]. However, it is fundamental to recognize that specific surgical techniques for CrCL rupture resolution, such as TPLO, generally change the stifle kinematics with a greater stress on patellar ligament. The increased stress on the patellar ligament after a TPLO can theoretically be explained by the variation of lever-arm lengths after osteotomies. The lever arm of the patellar ligament is the distance between the femorotibial contact point to the point of attachment of the patellar ligament to the tibial tuberosity. With a TPLO, this lever arm can be reduced by about 10% compared to an intact joint, and a shorter lever arm requires a greater force to extend the joint. The quadriceps muscle will generate this force, and this condition will result in greater traction and greater stress on the patellar ligament [32]. In this specific condition, exercises that lead to the increased flexion–extension of the knee joint and intensive underwater therapy (or especially swimming) should be avoided in the early phase after TPLO [172].

Recent studies describe innovative therapeutic approaches for the conservative management of CrCL rupture, especially in cases of partial rupture [208]. In 2016, Canapp and collaborators evaluated the use of autologous bone marrow aspirate concentrate (Bone Marrow Aspirate Concentrate: BMAC) or adipose-derived progenitor cells (Adipose-Derived Progenitor Cells: ADPC) combined with platelet-rich plasma (Platelet-Rich Plasma: PRP) to promote ligament regeneration, concluding that with both preparations, the outcome of the treatment is positive. Healing of the cranial cruciate ligament was confirmed by arthroscopy, and the improvement in joint function was assessed by gait analysis. The result was also optimal thanks to the association of a specific rehabilitation protocol and the use of the brace [153]. Rehabilitation goals in human medicine are the same as those described in veterinary medicine [160,161,165,167,209,210,211]. The main differences are the advantage of receiving direct feedback from the patient on the efficacy and side effects of the methods. Furthermore, while it is possible to combine exercises in closed and open kinetic chains for muscle strengthening [160,212,213,214], in dogs, the choice necessarily falls on the former, as it appears impossible to lead the dog to complete exercises that require free swinging of the injured limb. In addition, it is important to emphasize that in human medicine, part of the rehabilitation program can be conducted independently by the patient, unlike dogs, who requires continuous assistance from the therapist and/or owner.

## 6. Conclusions

Although cranial cruciate ligament rupture represents one of the most common orthopaedic diseases in veterinary and human medicine, its exact etiopathogenesis is still partially unknown. In dogs and humans, the femoral-tibio-patellar joint, due to its particular conformation and its strategic position at an intermediate level between hip and foot regions, plays a key static and dynamic role. In both species, by modern motion analysis techniques, which currently find greater application in human medicine, it is possible to investigate the biomechanical functions of the knee, which are largely overlapping, as well as the structural properties and functions of the cranial cruciate ligament. The main differences between the two species reside in weight distribution during while standing and while in motion, which is also supported by the forelimbs in dogs, and in the extent of the forces that break down at the joint level. In both species, however, the weight load translates into a force, called cranial tibial thrust, which pushes the tibia forward, stressing the cruciate ligament. This force is all the more decisive when the inclination of the tibial plateau is more evident; therefore, in the dog, in which greater values of this inclination are found, the ligament is more stressed and more likely to fall victim to degenerative processes. In dogs, progressive degeneration with structural weakening is the most accredited hypothesis to explain the onset of a lesion. Some of the factors responsible for this are to be found in the signalment of the animal (such as race, sex, and age), and others are related to the stifle conformation as well as to the development of periarticular muscle masses. Among all of these factors, the real contribution of genetics has yet to be fully clarified. In dogs, several studies have shown the association between ligament rupture and some genetic polymorphisms related to the genes responsible for the structural properties and strength of the ligament itself. More recent studies have identified single nucleotide polymorphisms affecting genomic loci, which are responsible for the angulation of the tibial plateau and the width of the tibial tuberosity, in some breeds, such as the Newfoundland and Labrador breeds. Further scientific studies need to be performed for a better understanding of the etiopathogenesis and inheritance of this lesion in human medicine as well. Although some of the factors responsible for ligament degeneration have also been investigated in human medicine, in humans, this injury generally occurs in a traumatic manner during participation in sporting activities that require jumps, sudden changes of direction, or where the player may come into contact with opponents. Traumatic rupture is possible also in veterinary medicine, but it is rare and is generally observed in sporting dogs of any breed and age. In human medicine, it has also been shown that women are more predisposed to the onset of this injury; this cannot only be attribute to hormonal reasons, and the reasons lie in numerous anatomical and biomechanical factors. In dogs, the gender difference has not been as widely discussed. Finally, it is important to emphasize the implementation of physiotherapeutic rehabilitation protocols in cases where conservative management of the partially or totally ruptured ligament has been chosen as well as in the post-operative phase of surgical resolution. In both species, the key points of rehabilitation programs are pain management, ROM restoration, periarticular and core muscles strengthening, and correction of proprioceptive deficits. The assistance of the therapist (and also the owner) is essential, especially for dogs, in order to guide the exercises during their execution. This aspect represents a substantial difference compared to human medicine, in which patients can complete an important part of the rehabilitation program independently. Furthermore, it must be emphasized that, while it is possible to combine closed and open kinetic chain muscle strengthening exercises in humans, in veterinary medicine, the choice necessarily falls on the former, as it is unthinkable to lead the dog to complete exercises that require free oscillation of injured limb. In conclusion, physiotherapy plays a key role in restoring the correct functionality of the injured knee and in increasing the probability of returning to normal daily and sporting activities. If this point has been well understood in human medicine, it is not always observed in veterinary clinic, as owners often neglect the importance of physiotherapy without considering the negative consequences on the quality of life of their animals.

## Figures and Tables

**Table 1:** Main predisposing factors for Cranial cruciate ligament (CrCL) rupture in dogs in comparison with humans.

Predisposing Factors	Dog	Man/Woman
Breed and body weight	Large breeds and/or overweight dogs	Obesity
Age	2–10 years old	15–45 years old
Sex	Neutered males and spayed females	Females > Males ^1^
Genetics	SNP affecting genes responsible for strength and structure of CrCL	Familiar predisposition with mutations in collagen genes
Stifle deformation	Low Intercondylar notch High Tibial plateau inclination Hyperextended position of the pelvic limbs Genu varum	Low Intercondylar notch High Tibial plateau inclination Valgus knee conformation

^1^ Mostly in athletes.

**Table 2:** Differences between dogs and humans for CrCL rupture in relation to gender (sex).

Gender	
Dog	Man/Woman
Higher incidence in neutered males and spayed females Increased activation of LH receptors High body weight and obesity Delay in tibia plate closure	Higher predisposition in women than in men Valgus conformation Hormonal factors Mutations in collagen genes

## Data Availability

All data are contained within this article.
